# Ethenzamide–gentisic acid–acetic acid (2/1/1)

**DOI:** 10.1107/S1600536810012407

**Published:** 2010-04-10

**Authors:** Srinivasulu Aitipamula, Pui Shan Chow, Reginald B.H. Tan

**Affiliations:** a Institute of Chemical and Engineering Sciences, A*STAR (Agency for Science, Technology and Research), 1 Pesek Road, Jurong Island, 627833 Singapore; b Department of Chemical and Biomolecular Engineering, National University of Singapore, 4 Engineering Drive, 117576 Singapore

## Abstract

In the title co-crystal solvate, 2-ethoxy­benzamide–2,5-dihydroxy­benzoic acid–ethanoic acid (2/1/1), 2C_9_H_11_NO_2_·C_7_H_6_O_4_·C_2_H_4_O_2_, two nonsteroidal anti-inflammatory drugs, ethenzamide (systematic name: 2-ethoxy­benzamide) and gentisic acid (systematic name: 2,5-dihydroxy­benzoic acid), together with acetic acid (systematic name: ethanoic acid) form a four-component mol­ecular assembly held together by N—H⋯O and O—H⋯O hydrogen bonds. This assembly features two symmetry-independent mol­ecules of ethenzamide, forming supra­molecular acid–amide heterosynthons with gentisic acid and acetic acid. These heterosynthons involve quite strong O—H⋯O [O⋯O = 2.5446 (15) and 2.5327 (15) Å] and less strong N—H⋯O [N⋯O = 2.9550 (17) and 2.9542 (17) Å] hydrogen bonds. The overall crystal packing features several C—H⋯O and π–π stacking inter­actions [centroid–centroid distance = 3.7792 (11) Å].

## Related literature

For information on three polymorphs of a 1:1 co-crystal involving ethenzamide and gentisic acid, see: Aitipamula *et al.* (2009*a* ▶). For other co-crystals of ethenzamide, see: Aitipamula *et al.* (2009*b* ▶); Moribe *et al.* (2004 ▶). For related information on the drug activity of ethenzamide, see: Hirasawa *et al.* (1999 ▶). For the crystal structure of ethenzamide, see: Pagola & Stephens (2009 ▶). For related information on the drug activity of gentisic acid, see: Lorico *et al.* (1986 ▶). For more information on the supra­molecular heterosynthons, see: Fleischman *et al.* (2003 ▶). For reviews on pharmaceutical co-crystals, see: Schultheiss & Newman (2009 ▶); Almarsson & Zaworotko (2004 ▶). For more information on the hydrogen bonding, see: Desiraju & Steiner (1999 ▶).
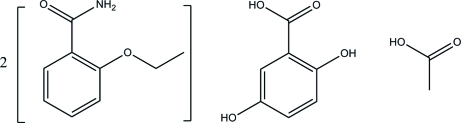


## Experimental

### 

#### Crystal data

2C_9_H_11_NO_2_·C_7_H_6_O_4_·C_2_H_4_O_2_
*M* *_r_* = 544.55
Triclinic, 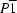

*a* = 8.8083 (18) Å
*b* = 8.8802 (18) Å
*c* = 19.880 (4) Å
α = 93.65 (3)°
β = 93.55 (3)°
γ = 119.45 (3)°
*V* = 1343.5 (6) Å^3^
*Z* = 2
Mo *K*α radiation
μ = 0.10 mm^−1^
*T* = 110 K
0.33 × 0.29 × 0.22 mm

#### Data collection

Rigaku Saturn CCD area-detector diffractometer
Absorption correction: multi-scan (Blessing, 1995 ▶) *T*_min_ = 0.967, *T*_max_ = 0.978
19296 measured reflections
6594 independent reflections
6074 reflections with *I* > 2σ(*I*)
*R*_int_ = 0.025

#### Refinement

*R*[*F* ^2^ > 2σ(*F* ^2^)] = 0.050
*wR*(*F* ^2^) = 0.135
*S* = 1.11
6594 reflections
380 parameters
H atoms treated by a mixture of independent and constrained refinement
Δρ_max_ = 0.25 e Å^−3^
Δρ_min_ = −0.23 e Å^−3^

### 

Data collection: *CrystalClear* (Rigaku, 2008 ▶); cell refinement: *CrystalClear*; data reduction: *CrystalClear*; program(s) used to solve structure: *SHELXS97* (Sheldrick, 2008 ▶); program(s) used to refine structure: *SHELXL97* (Sheldrick, 2008 ▶); molecular graphics: *X-SEED* (Barbour, 2001 ▶); software used to prepare material for publication: *SHELXTL* (Sheldrick, 2008 ▶) and *PLATON* (Spek, 2009 ▶).

## Supplementary Material

Crystal structure: contains datablocks global, I. DOI: 10.1107/S1600536810012407/fb2192sup1.cif

Structure factors: contains datablocks I. DOI: 10.1107/S1600536810012407/fb2192Isup2.hkl

Additional supplementary materials:  crystallographic information; 3D view; checkCIF report

## Figures and Tables

**Table 1:** Hydrogen-bond geometry (Å, °)

*D*—H⋯*A*	*D*—H	H⋯*A*	*D*⋯*A*	*D*—H⋯*A*
N1—H1⋯O2	0.926 (19)	1.941 (18)	2.6472 (19)	131.6 (14)
N1—H2⋯O5^i^	0.90 (2)	2.085 (18)	2.9550 (17)	163.0 (15)
N2—H7⋯O4	0.879 (18)	1.959 (17)	2.6536 (16)	135.0 (17)
N2—H10⋯O9^ii^	0.912 (18)	2.057 (17)	2.9542 (17)	167.4 (17)
O6—H11⋯O1^iii^	1.02 (2)	1.53 (2)	2.5327 (15)	167.0 (18)
O7—H16⋯O5	0.90 (2)	1.80 (2)	2.6183 (15)	149 (3)
O8—H19⋯O9^iv^	0.96 (2)	1.77 (2)	2.7231 (16)	173 (2)
O10—H20⋯O3^v^	0.99 (2)	1.56 (2)	2.5446 (15)	171 (2)
C8—H8*A*⋯O1^vi^	0.99	2.46	3.3768 (19)	154
C13—H13⋯O8^vii^	0.95	2.55	3.452 (2)	159
C14—H14⋯O10^viii^	0.95	2.53	3.348 (2)	145

Symmetry codes: (i) 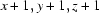; (ii) 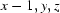; (iii) 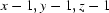; (iv) 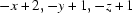; (v) 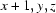; (vi) 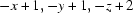; (vii) 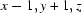; (viii) 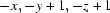.

**Fig. 1.**
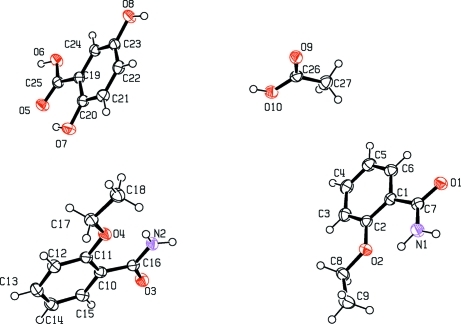
The title molecules of ethenzamide, gentisic acid and aceitic acid with the atom labels and 50% probability displacement ellipsoids for non-H atoms.

**Fig. 2.**
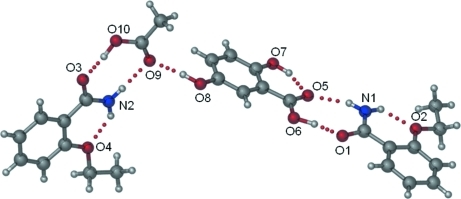
The hydrogen bonded four-component molecular assembly in the crystal structure of the title cocrystal solvate. Atoms participating in the hydrogen bonding were labelled.

**Fig. 3.**
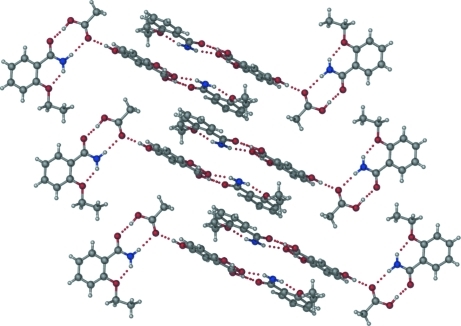
Section of the crystal structure, showing the π-π stacking interaction between the aromatic rings of the four-component molecular assemblies.

## supplementary crystallographic information

### Comment

Ethenzamide (2-ethoxybenzamide) belongs to a non-steroidal anti-inflammatory drug (NSAID) used mainly in combination with other ingredients for the treatment of mild to moderate pains (Hirasawa *et al.*, 1999). The crystal structure of ethenzamide has been recently solved using the high-resolution powder X-ray diffraction (Pagola & Stephens, 2009). Gentisic acid (2,5-dihydroxybenzoic acid) is also a NSAID (Lorico *et al.*, 1986).

Pharmaceutical cocrystals can be defined as molecular complexes formed between a neutral or ionic active pharmaceutical ingredient (API) and a pharmaceutically acceptable compound that is a solid under ambient conditions (Almarsson & Zaworotko, 2004). With our interest in pharmaceutical cocrystals and polymorphism, we recently reported three polymorphs of a 1:1 cocrystal involving ethenzamide and gentisic acid, and showed that the dissolution rates of the cocrystal polymorphs were improved twice when compared to that of the parent ethenzamide (Aitipamula *et al.*, 2009a).

In attempt to prepare pure polymorphs of a cocrystal involving ethenzamide and gentisic acid, they were cocrystallized in 1:1 molar ratio from several organic solvents. Whereas all the crystallization batches resulted in reported 1:1 cocrystal polymorphs (Aitipamula *et al.*, 2009a), crystallization from acetic acid yielded a solvate in which the ethenzamide, gentisic acid, and acetic acid were present in 2:1:1 molar ratio. We present here its crystal structure and analyze the hydrogen bonding.

The crystal structure contains two molecules of ethenzamide, one molecule of gentisic acid and one molecule of acetic acid in the asymmetric unit (Fig. 1). In the structure, gentisic acid and acetic acid molecules are engaged in the formation of acid-amide heterosynthons with symmetry independent molecules of ethenzamide involving quite strong O—H···O [O···O = 2.5446 (15) and 2.5327 (15) Å] and less strong N—H···O [N···O = 2.9550 (17) and 2.9542 (17) Å] hydrogen bonds (Table 1) (Desiraju & Steiner, 1999). The *anti*-N—H of the primary amide of ethenzamide and the 2-hydroxy group of gentisic acid form an intramolecular N—H···O [N···O = 2.6472 (19) and 2.6536 (16) Å] and O—H···O [O···O = 2.6183 (15)] hydrogen bonds, respectively (Table 1). Hydroxy atom of O8 of the gentisic acid acts as a hydrogen bond donor to atom O9 of the acetic acid at (2-*x*,1-*y*,1-*z*), and generates a four-component molecular assembly which involves two molecules of ethenzamide, one molecule each of gentisic acid and acetic acid (Fig. 2). It is worth mentioning that the solvent (acetic acid) molecule is an integral part of the four-component molecular assembly, which is bonded in the same way as the remaining constituents that participate in the heterosynthon formation. The four-component molecular assemblies are further stabilized in the crystal structure by various C—H···O interactions (Table 1) (Desiraju & Steiner, 1999), and by the π-π stacking interaction involving the phenyl rings of the molecules of ethenzamide and gentisic acid: *Cg1*···*Cg2* (1-*x*, 1-*y*, 1-*z*) = 3.7792 (11) Å, where *Cg1* and *Cg2* denote the centroids of the rings C1—C6 and C19—C24 of ethenzamide and gentisic acid, respectively (Fig. 3).

In the light of the overwhelming interest in the development of pharmaceutical cocrystals for improving the physico-chemical properties of the APIs (Schultheiss & Newman, 2009), the title cocrystal solvate reported here presents some special features. First, it contains two APIs and thus can be considered as a multi-API cocrystal. Second, it contains the pharmaceutically acceptable acetic acid in the crystal structure. These two aspects make the title cocrystal solvate a potential solid form for development of a combination drug involving ethenzamide and gentisic acid.

### Experimental

The title cocrystal solvate was obtained by slow evaporation of a glacial acetic acid (5 ml) solution of a 1:1 molar ratio of ethenzamide (100 mg, 0.605 mmol) and gentisic acid (93.3 mg, 0.605 mmol) at ambinent conditions. The block-shaped crystals, the dimensions of which were typically as those of the used sample for data collection, were obtained within 7 days.

### Refinement

Though all the H-atoms could be dinstinguished in the difference electron density map, the H-atoms bonded to C-atoms were included at the geometrically idealized positions and refined in riding-model approximation with C—H = 0.95 Å (aryl), 0.99 Å (methylene), and 0.98 Å (methyl). *Uiso*(H)_aryl/methylene_=1.2 *Ueq*(C) and *Uiso*(H)_methyl_=1.5 *Ueq*(C). The positional parameters of the H-atoms bonded to N and O were allowed to be refined freely while *Uiso*(H)_amine_=1.2 *Ueq*(N) and *Uiso*(H)_hydroxyl_=1.5 *Ueq*(O).

### Crystal data

**Table:**

2C_9_H_11_NO_2_·C_7_H_6_O_4_·C_2_H_4_O_2_	*Z* = 2
*M**_r_* = 544.55	*F*(000) = 576
Triclinic, *P*1	*D*_x_ = 1.346 Mg m^−^^3^
Hall symbol: -P 1	Mo *K*α radiation, λ = 0.71073 Å
*a* = 8.8083 (18) Å	Cell parameters from 3760 reflections
*b* = 8.8802 (18) Å	θ = 2.1–31.0°
*c* = 19.880 (4) Å	µ = 0.10 mm^−^^1^
α = 93.65 (3)°	*T* = 110 K
β = 93.55 (3)°	Block, yellow
γ = 119.45 (3)°	0.33 × 0.29 × 0.22 mm
*V* = 1343.5 (6) Å^3^	

### Data collection

**Table:**

Rigaku Saturn CCD area-detector diffractometer	6594 independent reflections
Radiation source: fine-focus sealed tube	6074 reflections with *I* > 2σ(*I*)
graphite	*R*_int_ = 0.025
ω scans	θ_max_ = 28.3°, θ_min_ = 2.1°
Absorption correction: multi-scan (Blessing, 1995)	*h* = −11→11
*T*_min_ = 0.967, *T*_max_ = 0.978	*k* = −11→9
19296 measured reflections	*l* = −26→24

### Refinement

**Table:**

Refinement on *F*^2^	Secondary atom site location: difference Fourier map
Least-squares matrix: full	Hydrogen site location: difference Fourier map
*R*[*F*^2^ > 2σ(*F*^2^)] = 0.050	H atoms treated by a mixture of independent and constrained refinement
*wR*(*F*^2^) = 0.135	*w* = 1/[σ^2^(*F*_o_^2^) + (0.0677*P*)^2^ + 0.2882*P*] where *P* = (*F*_o_^2^ + 2*F*_c_^2^)/3
*S* = 1.11	(Δ/σ)_max_ = 0.001
6594 reflections	Δρ_max_ = 0.25 e Å^−^^3^
380 parameters	Δρ_min_ = −0.23 e Å^−^^3^
0 restraints	Extinction correction: *SHELXL97* (Sheldrick, 2008), Fc^*^=kFc[1+0.001xFc^2^λ^3^/sin(2θ)]^-1/4^
Primary atom site location: structure-invariant direct methods	Extinction coefficient: 0.0054 (18)

### Special details

**Table:**

Geometry. All esds (except the esd in the dihedral angle between two l.s. planes) are estimated using the full covariance matrix. The cell esds are taken into account individually in the estimation of esds in distances, angles and torsion angles; correlations between esds in cell parameters are only used when they are defined by crystal symmetry. An approximate (isotropic) treatment of cell esds is used for estimating esds involving l.s. planes.
Refinement. Refinement of *F*^2^ against ALL reflections. The weighted *R*-factor wR and goodness of fit *S* are based on *F*^2^, conventional *R*-factors *R* are based on *F*, with *F* set to zero for negative *F*^2^. The threshold expression of *F*^2^ > σ(*F*^2^) is used only for calculating *R*-factors(gt) etc. and is not relevant to the choice of reflections for refinement. *R*-factors based on *F*^2^ are statistically about twice as large as those based on *F*, and *R*- factors based on ALL data will be even larger.

### Fractional atomic coordinates and isotropic or equivalent isotropic displacement parameters (Å^2^)

**Table:**

	*x*	*y*	*z*	*U*_iso_*/*U*_eq_	
O4	0.18598 (11)	0.96579 (12)	0.43017 (5)	0.0280 (2)	
O9	1.05478 (12)	0.54627 (13)	0.63748 (5)	0.0319 (2)	
O3	−0.22283 (12)	0.57234 (13)	0.51754 (5)	0.0324 (2)	
O10	0.76432 (12)	0.38996 (13)	0.61300 (5)	0.0316 (2)	
H20	0.781 (2)	0.464 (3)	0.5756 (10)	0.047*	
N2	0.06949 (14)	0.72419 (16)	0.51542 (6)	0.0281 (2)	
H10	0.079 (2)	0.668 (2)	0.5509 (9)	0.034*	
H7	0.159 (2)	0.811 (2)	0.5000 (9)	0.034*	
C16	−0.09157 (16)	0.68847 (16)	0.49396 (6)	0.0249 (2)	
C11	0.01615 (16)	0.92217 (16)	0.41021 (6)	0.0251 (3)	
C12	−0.02542 (17)	1.00581 (18)	0.36157 (7)	0.0295 (3)	
H12	0.0654	1.0958	0.3409	0.035*	
C18	0.49661 (17)	1.13984 (19)	0.43493 (7)	0.0325 (3)	
H18A	0.5037	1.1604	0.4843	0.049*	
H18B	0.5932	1.2403	0.4180	0.049*	
H18C	0.5050	1.0356	0.4229	0.049*	
C17	0.32440 (16)	1.11405 (17)	0.40346 (7)	0.0285 (3)	
H17A	0.3150	1.2191	0.4149	0.034*	
H17B	0.3155	1.0925	0.3535	0.034*	
C10	−0.11871 (16)	0.78677 (16)	0.44080 (6)	0.0246 (2)	
C15	−0.29227 (16)	0.74279 (17)	0.42099 (7)	0.0277 (3)	
H15	−0.3844	0.6526	0.4411	0.033*	
C13	−0.19922 (18)	0.95831 (18)	0.34313 (7)	0.0308 (3)	
H13	−0.2264	1.0162	0.3099	0.037*	
C26	0.91047 (17)	0.43317 (17)	0.65051 (7)	0.0283 (3)	
C14	−0.33339 (17)	0.82690 (18)	0.37295 (7)	0.0308 (3)	
H14	−0.4521	0.7951	0.3605	0.037*	
C27	0.8867 (2)	0.3342 (2)	0.71097 (8)	0.0371 (3)	
H27A	0.9874	0.3168	0.7199	0.056*	
H27B	0.7791	0.2209	0.7020	0.056*	
H27C	0.8780	0.4001	0.7505	0.056*	
O1	0.91975 (12)	0.71644 (12)	1.01611 (5)	0.0296 (2)	
O2	0.45825 (12)	0.67508 (13)	0.93005 (5)	0.0298 (2)	
C1	0.66698 (16)	0.57974 (16)	0.93614 (6)	0.0247 (2)	
N1	0.75912 (17)	0.84922 (16)	1.00868 (6)	0.0322 (3)	
H1	0.656 (2)	0.845 (2)	0.9926 (9)	0.039*	
H2	0.829 (2)	0.926 (2)	1.0438 (9)	0.039*	
C7	0.78960 (16)	0.72148 (16)	0.98983 (6)	0.0254 (2)	
C2	0.50360 (17)	0.55485 (16)	0.90874 (6)	0.0262 (3)	
C6	0.71780 (18)	0.46025 (17)	0.91414 (7)	0.0291 (3)	
H6	0.8278	0.4760	0.9321	0.035*	
C8	0.28786 (17)	0.65018 (19)	0.90704 (7)	0.0316 (3)	
H8A	0.1940	0.5434	0.9228	0.038*	
H8B	0.2710	0.6394	0.8569	0.038*	
C9	0.2832 (2)	0.8079 (2)	0.93678 (8)	0.0388 (3)	
H9A	0.3025	0.8181	0.9863	0.058*	
H9B	0.1687	0.7961	0.9232	0.058*	
H9C	0.3755	0.9123	0.9201	0.058*	
C4	0.44999 (19)	0.29481 (18)	0.84136 (7)	0.0344 (3)	
H4	0.3759	0.1974	0.8093	0.041*	
C3	0.39524 (18)	0.41077 (18)	0.86209 (7)	0.0314 (3)	
H3	0.2839	0.3921	0.8445	0.038*	
C5	0.61151 (19)	0.31929 (18)	0.86680 (7)	0.0330 (3)	
H5	0.6489	0.2402	0.8519	0.040*	
O8	0.70122 (13)	0.23351 (13)	0.25999 (5)	0.0327 (2)	
H19	0.784 (3)	0.317 (3)	0.2955 (10)	0.049*	
O6	0.13707 (13)	−0.09895 (13)	0.11728 (5)	0.0319 (2)	
H11	0.039 (3)	−0.166 (3)	0.0787 (10)	0.048*	
O5	−0.01505 (12)	0.03810 (13)	0.13600 (5)	0.0327 (2)	
O7	0.10781 (13)	0.29851 (13)	0.23160 (5)	0.0325 (2)	
H16	0.033 (3)	0.217 (3)	0.1981 (10)	0.049*	
C24	0.41450 (17)	0.13298 (16)	0.20904 (6)	0.0254 (2)	
H24	0.4210	0.0417	0.1842	0.030*	
C22	0.54594 (17)	0.38590 (17)	0.29022 (6)	0.0285 (3)	
H22	0.6432	0.4691	0.3207	0.034*	
C23	0.55542 (16)	0.25175 (17)	0.25389 (6)	0.0264 (3)	
C19	0.26205 (16)	0.14500 (16)	0.19962 (6)	0.0248 (2)	
C25	0.11617 (17)	0.02315 (16)	0.14875 (6)	0.0262 (3)	
C21	0.39528 (17)	0.39826 (17)	0.28208 (7)	0.0285 (3)	
H21	0.3894	0.4890	0.3076	0.034*	
C20	0.25202 (17)	0.27906 (17)	0.23681 (6)	0.0263 (3)	

### Atomic displacement parameters (Å^2^)

**Table:**

	*U*^11^	*U*^22^	*U*^33^	*U*^12^	*U*^13^	*U*^23^
O4	0.0206 (4)	0.0310 (5)	0.0326 (5)	0.0122 (4)	0.0044 (3)	0.0081 (4)
O9	0.0255 (4)	0.0373 (5)	0.0296 (5)	0.0130 (4)	0.0020 (3)	0.0044 (4)
O3	0.0221 (4)	0.0334 (5)	0.0383 (5)	0.0106 (4)	0.0038 (4)	0.0089 (4)
O10	0.0241 (4)	0.0338 (5)	0.0340 (5)	0.0119 (4)	0.0045 (4)	0.0054 (4)
N2	0.0215 (5)	0.0322 (6)	0.0288 (5)	0.0116 (4)	0.0025 (4)	0.0071 (4)
C16	0.0222 (5)	0.0254 (6)	0.0258 (6)	0.0112 (5)	0.0028 (4)	−0.0010 (4)
C11	0.0230 (5)	0.0271 (6)	0.0257 (6)	0.0136 (5)	0.0007 (4)	−0.0018 (5)
C12	0.0292 (6)	0.0305 (6)	0.0301 (6)	0.0160 (5)	0.0026 (5)	0.0035 (5)
C18	0.0246 (6)	0.0338 (7)	0.0383 (7)	0.0130 (5)	0.0055 (5)	0.0097 (6)
C17	0.0243 (6)	0.0285 (6)	0.0314 (6)	0.0115 (5)	0.0059 (5)	0.0068 (5)
C10	0.0234 (6)	0.0246 (6)	0.0251 (6)	0.0121 (5)	0.0010 (4)	−0.0025 (4)
C15	0.0234 (6)	0.0258 (6)	0.0308 (6)	0.0111 (5)	−0.0010 (5)	−0.0037 (5)
C13	0.0327 (7)	0.0301 (7)	0.0317 (6)	0.0185 (5)	−0.0046 (5)	−0.0006 (5)
C26	0.0293 (6)	0.0302 (6)	0.0275 (6)	0.0166 (5)	0.0049 (5)	0.0002 (5)
C14	0.0254 (6)	0.0301 (7)	0.0359 (7)	0.0149 (5)	−0.0050 (5)	−0.0040 (5)
C27	0.0446 (8)	0.0372 (8)	0.0330 (7)	0.0220 (6)	0.0083 (6)	0.0079 (6)
O1	0.0282 (4)	0.0290 (5)	0.0326 (5)	0.0159 (4)	−0.0005 (4)	−0.0008 (4)
O2	0.0302 (5)	0.0331 (5)	0.0305 (5)	0.0195 (4)	0.0020 (4)	0.0017 (4)
C1	0.0276 (6)	0.0228 (6)	0.0231 (6)	0.0117 (5)	0.0045 (4)	0.0048 (4)
N1	0.0356 (6)	0.0299 (6)	0.0338 (6)	0.0201 (5)	−0.0035 (5)	−0.0042 (5)
C7	0.0280 (6)	0.0238 (6)	0.0253 (6)	0.0130 (5)	0.0050 (4)	0.0054 (4)
C2	0.0299 (6)	0.0275 (6)	0.0235 (6)	0.0154 (5)	0.0062 (5)	0.0060 (5)
C6	0.0309 (6)	0.0281 (6)	0.0306 (6)	0.0162 (5)	0.0053 (5)	0.0039 (5)
C8	0.0275 (6)	0.0372 (7)	0.0342 (7)	0.0186 (6)	0.0047 (5)	0.0091 (5)
C9	0.0370 (7)	0.0420 (8)	0.0474 (8)	0.0261 (7)	0.0089 (6)	0.0101 (6)
C4	0.0375 (7)	0.0292 (7)	0.0298 (7)	0.0123 (6)	0.0017 (5)	−0.0008 (5)
C3	0.0299 (6)	0.0316 (7)	0.0290 (6)	0.0126 (5)	0.0018 (5)	0.0025 (5)
C5	0.0391 (7)	0.0288 (7)	0.0332 (7)	0.0188 (6)	0.0052 (5)	−0.0001 (5)
O8	0.0291 (5)	0.0363 (5)	0.0357 (5)	0.0198 (4)	−0.0039 (4)	−0.0006 (4)
O6	0.0344 (5)	0.0295 (5)	0.0336 (5)	0.0193 (4)	−0.0058 (4)	−0.0050 (4)
O5	0.0278 (5)	0.0350 (5)	0.0357 (5)	0.0176 (4)	−0.0032 (4)	−0.0034 (4)
O7	0.0290 (5)	0.0329 (5)	0.0383 (5)	0.0185 (4)	0.0006 (4)	−0.0026 (4)
C24	0.0287 (6)	0.0240 (6)	0.0249 (6)	0.0141 (5)	0.0025 (4)	0.0037 (4)
C22	0.0297 (6)	0.0260 (6)	0.0257 (6)	0.0110 (5)	0.0007 (5)	0.0016 (5)
C23	0.0260 (6)	0.0278 (6)	0.0266 (6)	0.0142 (5)	0.0021 (4)	0.0054 (5)
C19	0.0255 (6)	0.0235 (6)	0.0243 (6)	0.0113 (5)	0.0019 (4)	0.0037 (4)
C25	0.0271 (6)	0.0255 (6)	0.0266 (6)	0.0135 (5)	0.0029 (4)	0.0040 (5)
C21	0.0314 (6)	0.0254 (6)	0.0287 (6)	0.0142 (5)	0.0034 (5)	0.0012 (5)
C20	0.0269 (6)	0.0264 (6)	0.0270 (6)	0.0138 (5)	0.0045 (4)	0.0051 (5)

### Geometric parameters (Å, °)

**Table:**

O4—C11	1.3720 (15)	C1—C7	1.4965 (19)
O4—C17	1.4466 (16)	N1—C7	1.3256 (17)
O9—C26	1.2289 (17)	N1—H1	0.930 (19)
O3—C16	1.2555 (16)	N1—H2	0.90 (2)
O10—C26	1.3112 (17)	C2—C3	1.395 (2)
O10—H20	0.99 (2)	C6—C5	1.385 (2)
N2—C16	1.3269 (16)	C6—H6	0.9500
N2—H10	0.913 (18)	C8—C9	1.506 (2)
N2—H7	0.879 (18)	C8—H8A	0.9900
C16—C10	1.4924 (19)	C8—H8B	0.9900
C11—C12	1.3913 (19)	C9—H9A	0.9800
C11—C10	1.4159 (18)	C9—H9B	0.9800
C12—C13	1.3899 (18)	C9—H9C	0.9800
C12—H12	0.9500	C4—C5	1.386 (2)
C18—C17	1.5058 (18)	C4—C3	1.386 (2)
C18—H18A	0.9800	C4—H4	0.9500
C18—H18B	0.9800	C3—H3	0.9500
C18—H18C	0.9800	C5—H5	0.9500
C17—H17A	0.9900	O8—C23	1.3705 (16)
C17—H17B	0.9900	O8—H19	0.96 (2)
C10—C15	1.4012 (17)	O6—C25	1.3134 (16)
C15—C14	1.384 (2)	O6—H11	1.02 (2)
C15—H15	0.9500	O5—C25	1.2397 (16)
C13—C14	1.389 (2)	O7—C20	1.3622 (16)
C13—H13	0.9500	O7—H16	0.90 (2)
C26—C27	1.499 (2)	C24—C23	1.3798 (19)
C14—H14	0.9500	C24—C19	1.4007 (18)
C27—H27A	0.9800	C24—H24	0.9500
C27—H27B	0.9800	C22—C21	1.3849 (19)
C27—H27C	0.9800	C22—C23	1.3941 (19)
O1—C7	1.2523 (16)	C22—H22	0.9500
O2—C2	1.3644 (16)	C19—C20	1.4040 (18)
O2—C8	1.4444 (16)	C19—C25	1.4756 (19)
C1—C6	1.3963 (18)	C21—C20	1.3955 (19)
C1—C2	1.4112 (18)	C21—H21	0.9500
			
C11—O4—C17	117.67 (10)	O1—C7—N1	121.31 (12)
C26—O10—H20	113.5 (11)	O1—C7—C1	118.85 (12)
C16—N2—H10	116.3 (11)	N1—C7—C1	119.85 (12)
C16—N2—H7	119.0 (11)	O2—C2—C3	122.70 (12)
H10—N2—H7	123.8 (16)	O2—C2—C1	117.45 (11)
O3—C16—N2	121.07 (12)	C3—C2—C1	119.85 (12)
O3—C16—C10	119.00 (11)	C5—C6—C1	121.49 (13)
N2—C16—C10	119.93 (12)	C5—C6—H6	119.3
O4—C11—C12	122.18 (12)	C1—C6—H6	119.3
O4—C11—C10	117.69 (11)	O2—C8—C9	106.37 (12)
C12—C11—C10	120.13 (12)	O2—C8—H8A	110.5
C13—C12—C11	120.32 (13)	C9—C8—H8A	110.5
C13—C12—H12	119.8	O2—C8—H8B	110.5
C11—C12—H12	119.8	C9—C8—H8B	110.5
C17—C18—H18A	109.5	H8A—C8—H8B	108.6
C17—C18—H18B	109.5	C8—C9—H9A	109.5
H18A—C18—H18B	109.5	C8—C9—H9B	109.5
C17—C18—H18C	109.5	H9A—C9—H9B	109.5
H18A—C18—H18C	109.5	C8—C9—H9C	109.5
H18B—C18—H18C	109.5	H9A—C9—H9C	109.5
O4—C17—C18	107.57 (11)	H9B—C9—H9C	109.5
O4—C17—H17A	110.2	C5—C4—C3	120.78 (13)
C18—C17—H17A	110.2	C5—C4—H4	119.6
O4—C17—H17B	110.2	C3—C4—H4	119.6
C18—C17—H17B	110.2	C4—C3—C2	120.05 (13)
H17A—C17—H17B	108.5	C4—C3—H3	120.0
C15—C10—C11	117.89 (12)	C2—C3—H3	120.0
C15—C10—C16	116.73 (12)	C6—C5—C4	119.29 (13)
C11—C10—C16	125.37 (11)	C6—C5—H5	120.4
C14—C15—C10	121.93 (13)	C4—C5—H5	120.4
C14—C15—H15	119.0	C23—O8—H19	109.3 (12)
C10—C15—H15	119.0	C25—O6—H11	110.3 (11)
C14—C13—C12	120.47 (13)	C20—O7—H16	105.4 (13)
C14—C13—H13	119.8	C23—C24—C19	120.98 (12)
C12—C13—H13	119.8	C23—C24—H24	119.5
O9—C26—O10	122.84 (13)	C19—C24—H24	119.5
O9—C26—C27	122.78 (13)	C21—C22—C23	120.24 (12)
O10—C26—C27	114.38 (12)	C21—C22—H22	119.9
C15—C14—C13	119.25 (12)	C23—C22—H22	119.9
C15—C14—H14	120.4	O8—C23—C24	117.91 (12)
C13—C14—H14	120.4	O8—C23—C22	122.62 (12)
C26—C27—H27A	109.5	C24—C23—C22	119.46 (12)
C26—C27—H27B	109.5	C24—C19—C20	119.47 (12)
H27A—C27—H27B	109.5	C24—C19—C25	120.45 (12)
C26—C27—H27C	109.5	C20—C19—C25	120.02 (12)
H27A—C27—H27C	109.5	O5—C25—O6	122.74 (12)
H27B—C27—H27C	109.5	O5—C25—C19	121.85 (12)
C2—O2—C8	119.79 (11)	O6—C25—C19	115.40 (11)
C6—C1—C2	118.51 (12)	C22—C21—C20	120.82 (12)
C6—C1—C7	116.30 (12)	C22—C21—H21	119.6
C2—C1—C7	125.14 (12)	C20—C21—H21	119.6
C7—N1—H1	120.7 (11)	O7—C20—C21	117.69 (12)
C7—N1—H2	117.4 (12)	O7—C20—C19	123.28 (12)
H1—N1—H2	120.6 (16)	C21—C20—C19	119.02 (12)

### Hydrogen-bond geometry (Å, °)

**Table:**

*D*—H···*A*	*D*—H	H···*A*	*D*···*A*	*D*—H···*A*
N1—H1···O2	0.926 (19)	1.941 (18)	2.6472 (19)	131.6 (14)
N1—H2···O5^i^	0.90 (2)	2.085 (18)	2.9550 (17)	163.0 (15)
N2—H7···O4	0.879 (18)	1.959 (17)	2.6536 (16)	135.0 (17)
N2—H10···O9^ii^	0.912 (18)	2.057 (17)	2.9542 (17)	167.4 (17)
O6—H11···O1^iii^	1.02 (2)	1.53 (2)	2.5327 (15)	167.0 (18)
O7—H16···O5	0.90 (2)	1.80 (2)	2.6183 (15)	149 (3)
O8—H19···O9^iv^	0.96 (2)	1.77 (2)	2.7231 (16)	173 (2)
O10—H20···O3^v^	0.99 (2)	1.56 (2)	2.5446 (15)	171 (2)
C8—H8A···O1^vi^	0.99	2.46	3.3768 (19)	154
C13—H13···O8^vii^	0.95	2.55	3.452 (2)	159
C14—H14···O10^viii^	0.95	2.53	3.348 (2)	145

Symmetry codes: (i) *x*+1, *y*+1, *z*+1; (ii) *x*−1, *y*, *z*; (iii) *x*−1, *y*−1, *z*−1; (iv) −*x*+2, −*y*+1, −*z*+1; (v) *x*+1, *y*, *z*; (vi) −*x*+1, −*y*+1, −*z*+2; (vii) *x*−1, *y*+1, *z*; (viii) −*x*, −*y*+1, −*z*+1.
